# Stress Cardiomyopathy as a Complication of SARS-CoV-2 Infection

**DOI:** 10.7759/cureus.39264

**Published:** 2023-05-20

**Authors:** Filipa Rodrigues, Rui Flores, Maria João Vilela, Carolina Nogueira, Ana Rita Raposo, Catarina Vieira

**Affiliations:** 1 Internal Medicine, Hospital de Braga, Braga, PRT; 2 Cardiology, Hospital de Braga, Braga, PRT; 3 Physical Medicine and Rehabilitation, Hospital de Braga, Braga, PRT

**Keywords:** covid-19, takotsubo syndrome, stress cardiomyopathy, sars-cov-2, case report

## Abstract

The worldwide spread of severe acute respiratory syndrome coronavirus 2 (SARS-CoV-2) in early 2020 led to the coronavirus disease 2019 (COVID-19) pandemic. Acute lung diseases, such as COVID-19 pneumonia, can trigger stress cardiomyopathy, raising concerns about potential cardiovascular complications related to these diseases. The current case involved a 72-year-old man with SARS-CoV-2 infection who was experiencing dyspnea, desaturation, and oppressive retrosternal chest pain. On his admission to the hospital, an electrocardiogram demonstrated sinus tachycardia, negative T waves in leads V4-V6, and slight ST-segment elevation in the same precordial leads. The patient also had an increased troponin I value and worsening of his baseline respiratory failure, which required starting noninvasive ventilation. The echocardiogram showed moderately depressed left ventricular systolic function and apical ballooning. The echocardiographic changes resolved during hospitalization without directed therapeutic intervention. We diagnosed Takotsubo syndrome associated with SARS-CoV-2 infection; however, the pathophysiological disruption remains to be clarified.

## Introduction

Following the description of the first cases in China in late 2019, severe acute respiratory syndrome coronavirus 2 (SARS-CoV-2) infection spread worldwide in just a few months, instigating fear and anxiety and raising concern about health complications such as cardiovascular disease risk [1,2]. The exact mechanism by which COVID-19 increases the risk of cardiovascular disease remains under investigation, with several hypotheses focusing on immunological dysregulation (e.g., cytokine storm) and myocardial viral tropism [1,3]. Additionally, the cardiovascular impact of the drugs used to treat COVID-19 and associated comorbidities, such as hydroxychloroquine, azithromycin, tocilizumab, and remdesivir, is not fully understood, demonstrating that much remains to be learned about the cardiovascular manifestations and complications of SARS-CoV-2 infection [3].

## Case presentation

A 72-year-old man presented to the emergency department (ED) for acute-onset chest pain and dyspnea. At admission, the patient reported feeling extremely anxious because some of his relatives had tested positive for SARS-CoV-2 infection. During the night before his admission, the patient noticed desaturation on a portable oximeter that was resistant to higher oxygen flows. The patient grew increasingly dyspneic and anxious owing to his fear of a possible SARS-CoV-2 infection. No other respiratory symptoms, such as a productive cough, were noted, and he denied fever. The next morning, he experienced oppressive retrosternal chest pain that lasted a few hours. Another episode of chest pain later in the day led to him presenting to the ED.

Prior medical history was notable for arterial hypertension, hyperuricemia, and class D (according to the Global Initiative for Chronic Obstructive Lung Disease) chronic obstructive pulmonary disease with chronic hypercapnic respiratory failure. The patient quit smoking 12 years before, and his last admission was two years prior for respiratory decompensation. His current medications included chlorthalidone, tiotropium bromide, formoterol, fluticasone, and aminophylline. He was also under daily oxygen and nocturnal noninvasive ventilation. A suspected allergy to ciprofloxacin was noted.

The differential diagnosis included acute coronary syndrome, Takotsubo syndrome (TTS), acute myocarditis, acute pulmonary thromboembolism, and aortic dissection.

**Figure 1:**
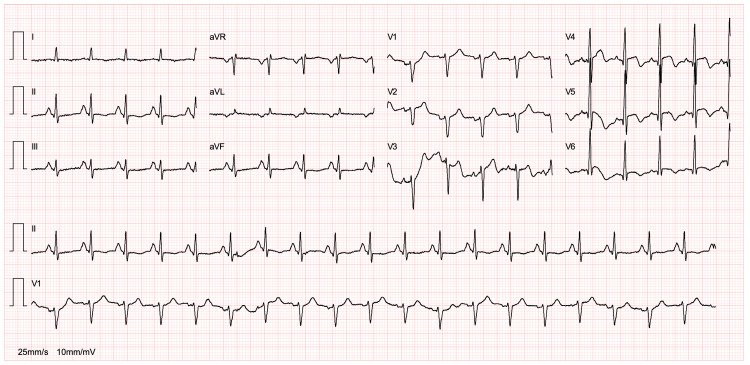
Electrocardiogram Electrocardiogram showing sinus tachycardia with 127 beats per minute, negative T waves, and ST-segment elevation in leads V4-6

At admission, the patient’s chest pain had resolved. Apart from obvious tachycardia, cardiac auscultation was normal. An electrocardiogram recorded at admission showed sinus tachycardia with 127 beats per minute, negative T waves in leads V4-6, and a discrete ST-segment elevation in the same precordial leads (Figure 1).

Blood tests showed an elevated level of troponin I of 7.0 ng/mL (normal range, <0.02 ng/mL). Additionally, the patient had a slightly elevated level of C-reactive protein and lymphopenia. An arterial blood sample showed mild to moderate deterioration of blood gas exchange, with respiratory acidosis, and the patient was put on noninvasive ventilation. Chest radiography showed bilateral perihilar infiltrates and a mild right pleural effusion. SARS-CoV-2 testing was positive, establishing the diagnosis of COVID-19. Chest computed tomography (CT) excluded signs of pneumonia but revealed paraseptal emphysema. Transthoracic echocardiography revealed a moderate depression of the left ventricle systolic function, with left ventricular ejection fraction (LVEF) of 35%, with hypokinesis of the apex and distal segments of all cardiac walls (Videos 1, 2).

**Video 1:** Transthoracic echocardiography (apical view) Transthoracic echocardiography (apical view) showing moderate depression of the left ventricle systolic function with hypokinesis of the apex and distal segments of all cardiac walls

**Video 2:** Transthoracic echocardiography (apical view) Transthoracic echocardiography (apical view) showing moderate depression of the left ventricle systolic function with hypokinesis of the apex and distal segments of all cardiac walls

Owing to the initial hypothesis of acute coronary syndrome, the patient was treated with double anti-platelet medications and started anticoagulation with low molecular weight heparin. Because the chest pain had resolved, cardiac catheterization was delayed. During hospitalization, troponin levels remained at a plateau (7.0 ng/mL) and were significantly dissociated from brain natriuretic peptide values, which were markedly elevated (3649 pg/mL). Echocardiographic changes progressively resolved throughout hospitalization. A transthoracic echocardiogram about two weeks later showed the normal systolic function of the left ventricle, with LVEF of 52% and normal-sized cardiac chambers (Video 3).

**Video 3:** Transthoracic echocardiography (apical view) Transthoracic echocardiography (apical view) showing total functional recovery and reversion of wall defects

No significant alterations in segmental contractibility were seen so cardiac angiography was postponed and antithrombotic drugs were suspended. Due to difficulties in weaning the patient from the ventilator, performing cardiac magnetic resonance imaging was not possible.

Corticosteroids were initiated for his lung disease, with significant improvement in blood gas exchange during hospitalization. Nevertheless, frequent episodes of desaturation occurred associated with moderate-intensity activity during hospitalization. Hydroxychloroquine and azithromycin were withheld because of concerns about cardiac toxicity. The patient was discharged after 26 days. He had two negative sets of nasopharyngeal swabs for SARS-CoV-2 prior to discharge, therefore fulfilling the criteria for cure defined by the hospital.

No readmissions were observed during the following three months.

## Discussion

We report the case of a 72-year-old man with COVID-19 who was admitted to the hospital with symptoms of TTS. TTS, which is also known as stress cardiomyopathy or broken heart syndrome, was first described in 1990 and was named based on ventriculography findings that resembled octopus-trapping pots in Japan [4]. TTS is frequently linked to strong emotional stimuli or vigorous physical stress, and it likely results from an exaggerated release of circulating catecholamines. However, its pathophysiology is widely debated [4]. Other hypotheses suggest epicardial vasospasm, microvascular dysfunction, and neurogenic myocardial stunning as potential causes or contributors [5,6]. Acute onset chest pain accompanied by dynamic ST-T segment deviations, elevated myocardial enzymes, and normal coronary angiography should prompt clinical investigation for TTS [4]. Echocardiographic and ventriculographic findings, such as apical ballooning with hypercontractility of the basal segments and transient left ventricular dysfunction, accompany the complex intricacies inherent to the diagnosis [4]. The acute and chronic management of this condition is not defined, reflecting that the underlying mechanisms are far from being understood [5].

The discussion of physiological mechanisms of diseases potentially related to COVID-19 is complex because distinguishing between random cardiac manifestations or myocardial lesions induced by the virus is difficult. However, acute lung diseases that cause respiratory failure, such as infections, are known potential triggers for stress cardiomyopathy [5]. Several SARS-CoV-2 pneumonia cases have been described as being related to TTS [7-10]. Our case highlights the possible viral tropism for cardiomyocytes, as no signs of pneumonia were present in the chest computed tomography. Nonetheless, the patient had severe and chronic respiratory lung disease, as well as worsening of his gas exchange and significant anxiety, which could translate to a higher risk for this condition per se. Other causes of chest pain, such as myocarditis, are possible and should be excluded by cardiac magnetic resonance imaging.

## Conclusions

The authors present the case of an elderly man diagnosed with COVID-19 and subsequently with echocardiographic findings suggestive of TTS, directing us to a correlation between these two entities. Not only is a possible viral tropism for cardiomyocytes highlighted, but also the contribution of severe chronic lung disease, severe respiratory failure, and significant anxiety as risk factors for the development of TTS.

This is a complex situation and awareness has increased regarding the cardiovascular and pulmonary sequelae of SARS-CoV-2 infection. Several studies are currently investigating laboratory and imaging markers. Their results should help determine whether acute manifestations of COVID-19 have negative impacts on cardiopulmonary function, which seems to be the likely case.
